# Modulatory Role of Simvastatin against Aluminium Chloride-Induced Behavioural and Biochemical Changes in Rats

**DOI:** 10.1155/2015/210169

**Published:** 2015-01-31

**Authors:** Madhavan Nampoothiri, Jessy John, Nitesh Kumar, Jayesh Mudgal, Gopalan Kutty Nampurath, Mallikarjuna Rao Chamallamudi

**Affiliations:** Department of Pharmacology, Manipal College of Pharmaceutical Sciences, Manipal University, Manipal, Karnataka 576104, India

## Abstract

*Objectives*. Aluminium, a neurotoxic agent in humans, has been implicated in the pathogenesis of neurodegenerative disorders. In this study, we examined the behavioral and biochemical effects of aluminium in rats with special emphasis on memory centres, namely, hippocampus and frontal cortex. Further, the effect of simvastatin treatment on aluminium intoxication was evaluated. *Methods*. Rats were exposed to aluminium chloride (AlCl_3_) for 60 days. Simvastatin (10 mg/kg/*p.o.*) and rivastigmine (1 mg/kg/*p.o.*) were administered daily prior to AlCl_3_. Behavioral parameters were assessed using Morris water maze test and actophotometer followed by biochemical investigations, namely, acetylcholinesterase (AChE) activity, TNF-*α* level, antioxidant enzymes (GSH, catalase), lipid peroxidation, and nitrite level in hippocampus and frontal cortex. Triglycerides, total cholesterol, LDL, and HDL levels in serum were also determined. *Key Findings*. Simvastatin treatment improved cognitive function and locomotor activity in rats. Simvastatin reversed hyperlipidemia and significantly rectified the deleterious effect of AlCl_3_ on AChE activity. Further, in hippocampus and frontal cortex, aluminium-induced elevation in nitrite and TNF-*α* and reduction in antioxidant enzymes were inhibited by simvastatin. *Conclusion*. To conclude, the present study suggests that simvastatin *per se* protects the neurons in hippocampus and frontal cortex from AlCl_3_, an environmental toxin.

## 1. Introduction

Aluminium is the most abundant metal on the earth. It gets access to the human body through drinking water, food, use of utensils, deodorants, and drugs. In the brain, aluminium accumulates in sensitive areas such as hippocampus and frontal cortex and is considered a potential contributing factor to the pathogenesis of neurodegenerative disorders like Alzheimer's disease (AD) and Parkinson's disease (PD) [1–3]. Aluminium-mediated neurodegeneration resulting in cognitive dysfunction has been associated with elevated amyloid precursor protein (APP) expression [4, 5], amyloid *β* (A*β*) deposition [6, 7], impaired cholinergic projections [8], apoptotic neuronal death [9, 10], and phosphorylated tau overexpression [11], which are also seen in AD patients. Thus, aluminium-induced cognitive deficit has been widely used for the preclinical testing of promising molecules against AD [12–14].

Current treatments only provide temporary and modest improvement in cognitive impairment and are considered symptomatic treatments. Thus, there is a need to develop novel and effective medication by focusing on alternative approaches, which go beyond acetylcholinesterase (AChE) inhibition and N-methyl-D-aspartate (NMDA) antagonism. Recent evidence indicates that prevalence of dementia is low among patients taking statins over a long period of time [15–17]. Contrary to this, there are clinical reports indicating that statins lead to cognitive impairment [18–20]. HMG-CoA reductase (3-hydroxy-3-methylglutaryl-coenzyme A-reductase) inhibitors (statins) are widely used as cholesterol-lowering agents. Besides cholesterol lowering, statins exert anti-inflammatory, antioxidant, and neuroprotective properties [21]. The literature also reports the potential of statins in modulating the amyloidogenic pathway [22, 23]. A USFDA report on February 28, 2012, states that statins produce reversible dementia in some population. However, since these are isolated and rare observations, FDA advised patients to continue their statin medication to prevent cardiac damage. Thus, there is no clarity till today on the role of statins in dementia. In view of this, there is scope for the prospective investigation to ascertain the pharmacological actions of statins in the area of memory impairment and cognitive dysfunction. Among different statins, we have selected simvastatin on the basis of its high lipophilicity and ease of traversing the blood brain barrier [24]. To date, there are no reports on simvastatin against aluminium-mediated behavioural and biochemical changes in rats. Thus, the study was taken up to investigate the role of lipid lowering drug (simvastatin) in spatial memory and to elicit the possible mechanisms through which it modulates the cognitive deficit in aluminium exposed rats.

## 2. Materials and Methods

### 2.1. Animals

Ninety-day-old male Wistar rats, weighing 200–250 g, were used for the study. The animals were obtained from Central Animal Research Facility (CARF) of Manipal University, Manipal. For seven days prior to the experiment, the animals were acclimatized to laboratory conditions and were maintained under controlled temperature (23 ± 2°C) and humidity (50 ± 5%) conditions. Standard conditions of 12 h light/dark cycle were provided. The animals were kept in sanitized polypropylene cages having sterile paddy husk as bedding with free access to food and water. The experimental protocol was approved by the Institutional Animal Ethics Committee, Kasturba Medical College, Manipal (IAEC/KMC/73/2012), and was carried out in accordance with the guidelines given by the Committee for the Purpose of Control and Supervision of Experiments on Animals (CPCSEA), Government of India.

### 2.2. Drugs and Chemicals

Acetylthiocholine iodide, reduced glutathione, thiobarbituric acid, griess reagent, hydrogen peroxide, and 5,5′-dithiobis-(2-nitrobenzoic acid) (DTNB) were obtained from Sigma-Aldrich Co. LLC (St. Louis, MO, USA). AlCl_3_ (Spectrochem Pvt. Limited, India), simvastatin (Biocon Ltd., India), and rivastigmine (Dr. Reddy's Laboratories, Hyderabad, India) were obtained. All chemicals used in this study were of analytical grade.

### 2.3. Experimental Design

On probe day (day 5), the animals were randomized based on ELT (escape latency) and divided into 6 groups (*n* = 8). AlCl_3_, simvastatin, and rivastigmine solutions were made freshly each day for administration. Rats were administered with AlCl_3_ (175 mg/kg) orally from day 6 (i.e., 24 h after the completion of retention trial on day 5) for 60 days. Aluminium chloride was dissolved in distilled water and administered orally at a dose of 0.5 mL/100 g bodyweight. The dose of AlCl_3_ was selected based on the previous literature reports [25, 26] and from a pilot study conducted. This dose was found to have a faster rate of induction with low incidence of mortality. Simvastatin (10 mg/kg) and the standard drug (rivastigmine 1 mg/kg), suspended in 0.5% sodium carboxy methyl cellulose (CMC), were given orally, 45 minutes before AlCl_3_ administration for 60 days beginning on day 6. The dose selection was based on the previous literature reports [27–29]. Bodyweights of the animals were taken on daily basis throughout the study. The groups were as follows.

 Group 1: normal control—received distilled water (5 mL/kg* p.o.*).
 Group 2: vehicle control—received 0.5% CMC (5 mL/kg* p.o.*).
 Group 3: AlCl_3_ (175 mg/kg* p.o.*).
 Group 4: rivastigmine (1 mg/kg* p.o.*) + AlCl_3_ (175 mg/kg* p.o.*).
 Group 5: simvastatin (10 mg/kg* p.o.*) + AlCl_3_ (175 mg/kg* p.o.*).

### 2.4. Spatial Memory Assessment Using Morris Water Maze [30]

The water maze contained a circular water pool with 150 cm diameter and 40 cm height.

The water pool was divided into northeast (NE), southeast (SE), southwest (SW), and northwest (NW) equally spaced quadrants along the circumference of the pool. In the NW quadrant, an escape platform (10 cm diameter) was kept, 2 cm below the water surface. Throughout the acquisition trials the platform was maintained in a constant location in NW quadrant. The rats were trained to locate this hidden platform. If the rat failed to find the platform within 60 sec, it was gently guided to the platform and was allowed to stay there for 15 sec. Animals had four acquisition trials per day for four consecutive days. To eliminate the quadrant effects, animal was positioned in each quadrant during each trial. Animals that failed to reach the platform in 20 s on the 4th trial day were excluded from the study. On probe day (day 5), 24 h after the last acquisition trial, escape platform was removed and retention trial was conducted. The animals were allowed to swim for 60 s before the end of session. Retention trials were repeated on day 65 on all groups to evaluate the memory consolidation. Data were obtained through a video camera attached to a computerized tracking system (Any Maze, Ugo Basile, Italy) fixed above the centre of the pool. Time to reach hidden platform (escape latency), latency to find the target quadrant (northwest latency), and percentage of time spent in target quadrant (NW) were measured during retention trials.

### 2.5. Locomotor Activity

Locomotor activity was assessed in animals using a digital photoactometer (INCO, Ambala, India). The ambulatory movements were recorded for a period of 10 min and expressed in terms of total photo beam counts for 10 min per animal [31]. Locomotor activity was assessed on 5th and 65th days before probe trial in Morris water maze.

### 2.6. Biochemical Evaluation

Animals were mildly anaesthetized at the end of the experimental period with diethyl ether and the blood samples were collected by retroorbital sinus puncture into microcentrifuge tubes.

Blood was allowed to clot for half an hour and then centrifuged at 10000 rpm for 10 minutes to obtain serum. Samples were divided into aliquots and stored at −20°C until biochemical analysis.

The triglyceride (GPO-POD method), total cholesterol (CHOD-POD method), LDL, and HDL levels were estimated in samples by end point method as per the manufacturer's instructions with the help of diagnostic kits (Aspen Laboratories, Mumbai) using ELISA plate reader.

### 2.7. Dissection and Tissue Preparation

Immediately after the probe trial on day 65, the animals were sacrificed by decapitation. On the dorsal side of the skull, an incision was made to expose and remove the brain rapidly from each rat. According to the method described by Glowinski and Iversen [32], hippocampus and frontal cortex were dissected. A 10% w/v homogenate of samples was prepared in ice-cold 0.1 M phosphate buffer pH 7.4 using an Ultra-Turrax T25 homogenizer at a speed of 9500 rpm.

### 2.8. Estimation of Acetylcholinesterase (AChE) Activity

The acetylcholinesterase activity was determined quantitatively by Ellman's method [33]. The enzyme activity was measured by following the increase of yellow colour produced from the reaction of thiocholine with 5,5′-dithiobis-(2-nitrobenzoic acid) [DTNB].

### 2.9. Measurement of Endogenous Antioxidant Defence System

The homogenate was used for the estimation of nitrite [34], lipid peroxidation [35], catalase activity [36], and glutathione [37] in hippocampus and frontal cortex.

### 2.10. Estimation of Total Protein

Total protein was estimated in all tissue samples by using Pierce BCA Protein Assay Kit as per the experimental protocol given by Thermo Scientific, USA.

### 2.11. Tumor Necrosis Factor-*α* (TNF-*α*) Estimation in Hippocampus

In the supernatant of the tissue homogenate, level of TNF-*α* was estimated by rat TNF-*α* enzyme linked immunosorbant assay (ELISA) kit as per the manufacturer's instruction given by Invitrogen Corporation, USA.

### 2.12. Statistical Analysis

Data were analyzed by one-way analysis of variance (ANOVA) followed by Tukey's post hoc test. Data were expressed as mean ± standard error of the mean and the values of *P* < 0.05 were considered statistically significant.

## 3. Results

### 3.1. Simvastatin Attenuated AlCl_3_-Induced Spatial Memory Deficit in Rats

**Figure 1:**
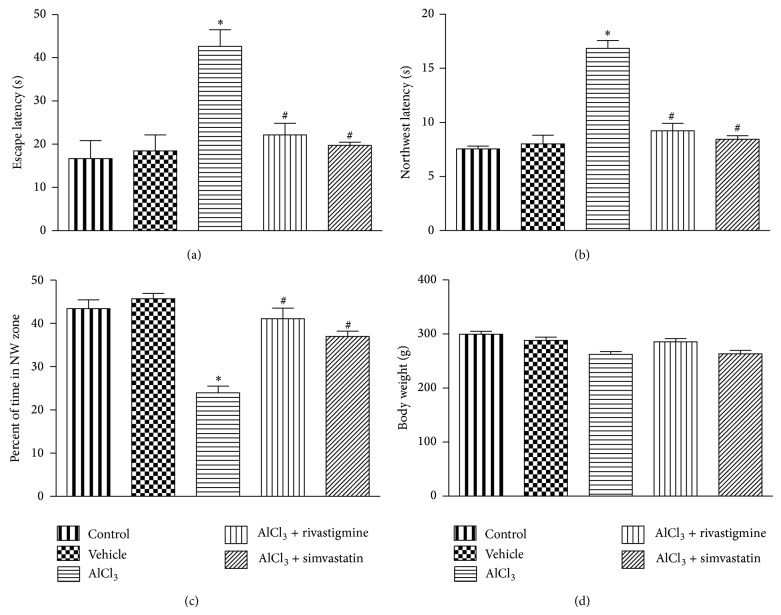
Effect of AlCl_3_ and AlCl_3_ + treatments on (a) escape latency time (latency to reach platform), (b) northwest latency, (c) percent of time spent in target quadrant (NW), and (d) body weight during retention trial after treatment. Data are presented as mean ± SEM (*n* = 8). ^*^ *P* < 0.05 as compared to control group and ^**#**^ *P* < 0.05 as compared to AlCl_3_ treated group.

After 60 days of AlCl_3_ intoxication in rats, significant spatial memory impairment was observed in Morris water maze test during the retention trial conducted on the 60th day (Figure 1). AlCl_3_ (175 mg/kg* p.o.*) treatment significantly (*P* < 0.05) raised ELT (Figure 1(a)) and NW latency (Figure 1(b)). During the probe trial on day 60, aluminium treated animals were found to spend significantly less time in the target quadrant (NW) than the control group (Figure 1(c)). Throughout the study, no significant change was found in the body weights among the treated groups (Figure 1(d)). The spatial memory deficit caused by AlCl_3_ was significantly (*P* < 0.05) reversed by simvastatin and rivastigmine (Figures 1(a), 1(b), and 1(c)).

### 3.2. Effect of Simvastatin on Locomotor Activity of Rats

**Figure 2:**
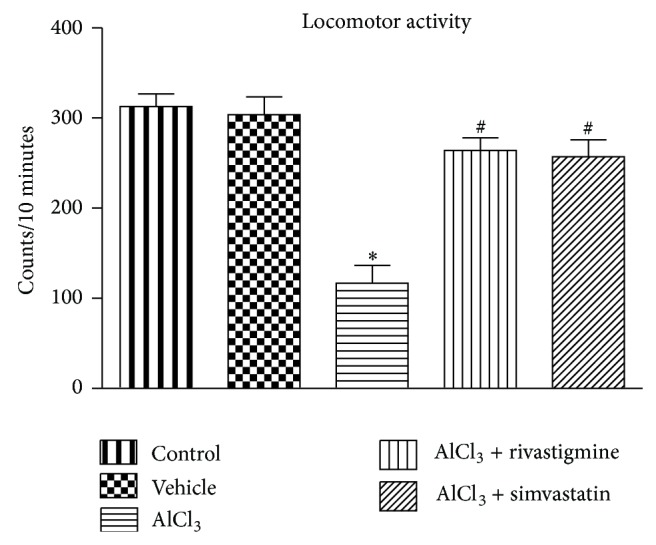
Effect of AlCl_3_ and AlCl_3_ + treatments on locomotor activity after treatment. Data are presented as mean ± SEM (*n* = 8). ^*^ *P* < 0.05 as compared to control group and ^**#**^ *P* < 0.05 as compared to AlCl_3_ treated group.

As shown in Figure 2, a significant reduction in the locomotor activity was observed in the animals of AlCl_3_ treated group after 60 days of treatment as compared to control animals. On the 65th day, the drug treatments (simvastatin and rivastigmine) significantly (*P* < 0.05) produced increased locomotor activity of animals as compared with AlCl_3_ group.

### 3.3. Simvastatin Reversed AlCl_3_-Induced Lipid Profile Alterations in Rats

AlCl_3_ produced significant (*P* < 0.05) upsurge in total cholesterol, triglycerides, and low-density lipoprotein (LDL) levels and significantly decreased serum high-density lipoprotein (HDL) levels compared with control group (Table 1). Simvastatin-treated group, but not the rivastigmine-treated group, reversed the elevation in lipid profile induced AlCl_3_ (Table 1).

### 3.4. Reversal of Diminished Acetylcholinesterase Activity by Simvastatin

Sixty days of chronic AlCl_3_ exposure significantly reduced AChE activity in the frontal cortex (*P* < 0.05) and hippocampus (*P* < 0.05) of rats as compared to normal control. In hippocampus and frontal cortex, AChE activity was significantly (*P* < 0.05) lowered by rivastigmine compared to control group. The effect of AlCl_3_ on AChE activity was significantly inhibited by 10 mg/kg simvastatin (Tables 2 and 3).

### 3.5. Attenuation of Elevated MDA and Nitrite Level by Simvastatin

In aluminium treated rats, levels of malondialdehyde (MDA) and nitrite in hippocampus and frontal cortex recorded a significant increase as compared with control group. Both rivastigmine and simvastatin (10 mg/kg) significantly reversed the elevated MDA and nitrite levels. Interestingly, simvastatin at 10 mg/kg showed a better reduction of MDA levels than rivastigmine in hippocampus region (Tables 2 and 3).

### 3.6. Elevation of Antioxidant Enzyme (Catalase, GSH) Levels by Simvastatin

The hippocampus and frontal cortex of the AlCl_3_ treated rats showed significant (*P* < 0.05) decrease in catalase and glutathione activity. Rivastigmine (1 mg/kg) and simvastatin (10 mg/kg) significantly improved the catalase and glutathione (GSH) levels as compared to AlCl_3_ treated group (Tables 2 and 3).

### 3.7. Simvastatin Inhibited TNF-*α* Level in Hippocampus of Rats

**Figure 3:**
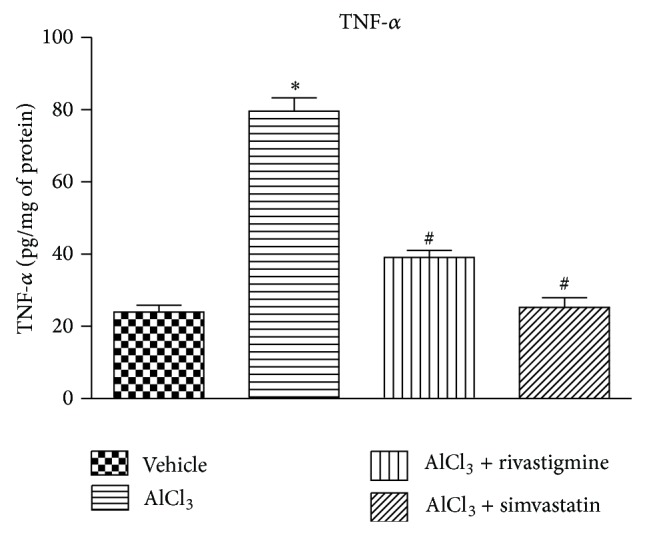
Effect of AlCl_3_ and AlCl_3_ + treatments on TNF-*α* level in the hippocampus of rats. Data are presented as mean ± SEM (*n* = 8). ^*^ *P* < 0.05 as compared to control group and ^**#**^ *P* < 0.05 as compared to AlCl_3_ treated group.

A threefold increase in TNF-*α* level was found in AlCl_3_ treated rats, whereas simvastatin (10 mg/kg) and rivastigmine significantly (*P* < 0.05) inhibited this rise in TNF-*α* levels (Figure 3).

## 4. Discussion

The study explores the protective effect of HMG Co-A reductase inhibitor, simvastatin, on AlCl_3_-induced behavioural and neurochemical changes in rats. After chronic exposure, aluminium accumulates in all brain regions with greater accumulation in cortex and hippocampus [38, 39]. Hippocampus and frontal cortex play an important role in learning and memory [40], which is severely affected in neurodegenerative disorders such as AD and PD.

Chronic aluminium exposure has been reported to result in cognitive [41] and locomotor impairment [42]. The cognitive deficit is evident from declined performance in Morris water maze test [43], passive avoidance task [44], and radial arm maze test [41]. In our study, aluminium treated rats displayed behavioural alterations, which are consistent with the previous reports. In water maze test, AlCl_3_ treatment resulted in behavioural changes such as spatial memory deficit, indicated by increased escape latency, northwest latency, and decreased percentage of time spent in NW zone. Rivastigmine (1 mg/kg) and simvastatin (10 mg/kg) antagonized the spatial memory deficit caused by aluminium. This suggests the neuroprotective role of simvastatin in correcting cognitive dysfunction associated with aluminium exposure. Throughout the treatment period no significant changes were observed in body weight and health status of animals.

Assessment of locomotor activity is a requirement for evaluating any possible CNS depressant/stimulant effect of interventions on animals. Similar to previous reports [42], a decline in locomotor activity in aluminium treated rats was observed, indicating the CNS depressant effect of chronic aluminium exposure. Thus, the overall cognitive behavioural changes by aluminium intoxication could be interlinked to locomotor impairment due to CNS depression. However, the impaired cholinergic transmission, oxidative/nitrergic stress, neuroinflammation, and dyslipidemia observed in aluminium exposed rats suggest that the cognitive deficit is the compounding effect of the above biochemical changes along with locomotor incoordination. Treatment with rivastigmine and simvastatin corrected the locomotor incoordination caused by AlCl_3_. These findings imply the ability of simvastatin to correct the aluminium-mediated behavioural changes.

Impaired cholinergic transmission is one of the factors implicated in the etiopathogenesis of memory deficit in AD. The neurodegeneration in frontal cortex and hippocampus areas of the brain [45] resulting in impaired cholinergic transmission occurs by two ways. Firstly, in AD patients, it occurs either due to (i) decline in acetylcholine (ACh) release or due to (ii) decreased choline acetyltransferase activity, which results in the scarcity of ACh [46–48]. Secondly, elevated acetylcholinesterase (AChE) enzyme further adds to scarcity of ACh at synapse by degrading the available ACh. This degradation of ACh is abolished by rivastigmine (AChE inhibitor) and, thus, is found effective in AD through improvement in cholinergic transmission. On the contrary, the literature also reports the reduced acetylcholinesterase activity in AD patients as compared with the normal [49, 50].

In the present study, AlCl_3_ inhibited acetylcholinesterase activity. Aluminium is a metal known to cause oxidative stress. Generally, oxidative stress has got the highest ability to cause damage to the highly active enzymes. Perhaps this property of AlCl_3_ might have produced the anticholinesterase activity observed in the present study with AlCl_3_. This observation corroborates with earlier workers [51, 52] who observed a biphasic response, namely, rise in AChE activity in short phase and a marked decline in AChE enzyme activity on long term administration. In consistence with the above reports, the present work demonstrated the second phase, that is, decline in AChE enzyme activity after chronic administration (60 days) of aluminium to rats. We assume that decreased AChE activity is due to the slow accumulation of aluminium in the brain and formation of aluminium complex with high affinity for the anionic site of enzyme [51, 52], leading to induction of oxidative stress to inhibit AChE enzyme activity. However, it is intriguing how an agent (AlCl_3_) observed to have anticholinesterase activity should cause AD. We hypothesize that AlCl_3_ is a neurotoxin; therefore it has the propensity to cause impaired cholinergic transmission by affecting the synthesis and release. These effects are more pronounced than AChE inhibition which may be partial. This is true because the AChE inhibitory activity of standard drug rivastigmine in our study was found to be more significant than AlCl_3_ activity. On the other hand, simvastatin did not possess any acetylcholinesterase inhibition mechanism in vivo but abolished the effect of aluminium on acetylcholinesterase enzyme.

Elevated total serum cholesterol and LDL-associated cholesterol have been implicated in AD patients [53–55]. Even though the brain cholesterol is independent of the peripheral cholesterol stores, several reports suggest the correlation between elevated peripheral cholesterol and AD. In the present study, aluminium exposed rats showed elevated levels of total cholesterol and LDL and decreased HDL levels in serum, which was found to be correlated with cognitive dysfunction. The dyslipidemia may be due to aluminium accumulation in the liver as reported earlier [56, 57]. As expected, chronic treatment of aluminium exposed rats with simvastatin, a HMG-CoA reductase inhibitor, reversed the altered lipid profile. These results suggest the ameliorative role of simvastatin in dementia through correction of dyslipidemia. However, rivastigmine failed to correct the altered lipid profile parameters induced by aluminium.

The literature reports the contribution of oxidative stress and neuroinflammation to the pathogenesis of neurodegenerative disorders [58, 59]. In AlCl_3_-induced cognitive dysfunction model, elevation of reactive oxygen species (ROS) level due to significant impairment of antioxidant enzyme system has been reported [43]. In the present study, AlCl_3_ treatment resulted in decrease in the levels of endogenous antioxidant enzymes like catalase and GSH in hippocampus and frontal cortex regions. Further, elevation of nitrite and malondialdehyde levels was also observed in these regions of aluminium exposed rats. The alterations in the antioxidant defence system like catalase, GSH, nitrite, and MDA levels were brought to normal by both rivastigmine and simvastatin treatment. Aluminium exposure augmented the elevation in hippocampus TNF-*α*, a proinflammatory mediator. The elevated hippocampus TNF-*α* stimulates microglia to release glutamate, which causes excitotoxicity [59, 60]. The rise in TNF-*α* was counteracted by treatment with rivastigmine and simvastatin. These results indicated the potential of simvastatin in preventing oxidative stress and neuroinflammation, thereby correcting the cognitive dysfunction. Future studies will be directed to study the role of TNF-*α* in frontal cortex along with hippocampus to link the possible mechanism of neuroinflammation mediated cognitive dysfunction.

## 5. Conclusion

Simvastatin exerted neuroprotective action against AlCl_3_-induced behavioural parameters such as cognitive deficit and locomotor impairment. Further, aluminium-mediated biochemical changes were reversed, where simvastatin was able to correct hyperlipidemia, oxidative stress, and neuroinflammation in hippocampus and cortex regions. Further studies are warranted to explore the link between AlCl_3_-mediated hyperlipidemia and associated dementia to establish the role of simvastatin in neuronal disturbances.

## Figures and Tables

**Table 1:** Effect of simvastatin on serum lipid profile.

Treatment	Total cholesterol	Triglycerides	LDL	HDL
	(mg/dL)	(mg/dL)	(mg/dL)	(mg/dL)
Normal control	68.63 ± 2.77	66.67 ± 1.93	21.15 ± 2.27	43.03 ± 1.10
Vehicle control	62.25 ± 4.36	65.21 ± 3.36	19.95 ± 2.19	44.65 ± 1.48
AlCl_3_	90.19 ± 3.22^*^	110.9 ± 9.85^*^	31.81 ± 2.78^*^	30.19 ± 0.98^*^
AlCl_3_ + rivastigmine	72.91 ± 3.99	72.45 ± 7.58	22.21 ± 3.17	31.68 ± 2.00
AlCl_3_ + simvastatin	55.12 ± 3.54^#^	60.81 ± 2.04^#^	11.76 ± 2.37^#^	42.12 ± 3.27^#^

Effect of AlCl_3_ and AlCl_3_ + treatments on (a) total cholesterol, (b) triglycerides, (c) LDL, and (d) HDL. Data are presented as mean ± SEM (*n* = 8). ^*^ *P* < 0.05 as compared to control group and ^#^ *P* < 0.05 as compared to AlCl_3_ treated group.

**Table 2:** Effect of simvastatin on AlCl_3_-induced biochemical changes on frontal cortex.

Treatment	MDA (nmol/mg of protein)	Nitrite (nmol/mg of protein)	Reduced glutathione (*μ*mol/mg of protein)	Catalase (*μ*mol of H_2_O_2_ decomposed/min/mg of protein)	AChE (*μ*moles of acetylthiocholine iodide hydrolyzed/min/mg of protein)
Normal control	0.085 ± 0.0129	1.71 ± 0.248	0.117 ± 0.0075	35.29 ± 1.64	0.0292 ± 0.0018
Vehicle control	0.074 ± 0.0070	1.87 ± 0.098	0.114 ± 0.0053	36.35 ± 2.61	0.0281 ± 0.0017
AlCl_3_	0.209 ± 0.0047^*^	3.75 ± 0.102^*^	0.067 ± 0.0022^*^	6.97 ± 0.38^*^	0.0141 ± 0.0014^*^
AlCl_3_ + rivastigmine	0.126 ± 0.0077^#^	2.32 ± 0.170^#^	0.104 ± 0.0031^#^	28.38 ± 3.18^#^	0.0094 ± 0.0021^#^
AlCl_3_ + simvastatin	0.125 ± 0.0225^#^	2.06 ± 0.073^#^	0.101 ± 0.0026^#^	24.39 ± 4.51^#^	0.0285 ± 0.0022^#^

Effect of AlCl_3_ and AlCl_3_ + treatments on frontal cortex (a) MDA, (b) nitrite level, (c) glutathione (GSH) level, (d) catalase activity, and (e) acetylcholinesterase activity. Data are presented as mean ± SEM (*n* = 8). ^*^ *P* < 0.05 as compared to control group and ^#^ *P* < 0.05 as compared to AlCl_3_ treated group.

**Table 3:** Effect of simvastatin on AlCl_3_-induced biochemical changes on hippocampus.

Treatment	MDA (nmol/mg of protein)	Nitrite (nmol/mg of protein)	Reduced glutathione (*μ*mol/mg of protein)	Catalase (*μ*mol of H_2_O_2_ decomposed/min/mg of protein)	AChE (*μ*moles of acetylthiocholine iodide hydrolyzed/min/mg of protein)
Normal control	0.091 ± 0.0138	1.64 ± 0.090	0.110 ± 0.0036	22.80 ± 1.57	0.0251 ± 0.0016
Vehicle control	0.091 ± 0.0073	1.87 ± 0.373	0.099 ± 0.0036	23.40 ± 1.71	0.0261 ± 0.0017
AlCl_3_	0.206 ± 0.0106^*^	3.86 ± 0.137^*^	0.053 ± 0.0010^*^	4.77 ± 0.61^*^	0.0074 ± 0.0021^*^
AlCl_3_ + rivastigmine	0.143 ± 0.0119^#^	1.95 ± 0.249^#^	0.084 ± 0.0043^#^	15.50 ± 1.43^#^	0.0060 ± 0.0011^#^
AlCl_3_ + simvastatin	0.182 ± 0.0114^#^	1.96 ± 0.435^#^	0.086 ± 0.0044^#^	12.11 ± 1.26^#^	0.0230 ± 0.0016^#^

Effect of AlCl_3_ and AlCl_3_ + treatments on hippocampus (a) MDA, (b) nitrite level, (c) glutathione (GSH) level, (d) catalase activity, and (e) acetylcholinesterase activity. Data are presented as mean ± SEM (*n* = 8). ^*^ *P* < 0.05 as compared to control group and ^#^ *P* < 0.05 as compared to AlCl_3_ treated group.
